# Predictors of Colorectal Cancer Screening among Average and High-Risk Saudis Population

**DOI:** 10.3390/jpm12050662

**Published:** 2022-04-20

**Authors:** Fuad H. Abuadas, Abdalkarem F. Alsharari, Mohammad H. Abuadas

**Affiliations:** 1 Nursing Department, College of Applied Medical Sciences, Jouf University, Sakaka 72388, Saudi Arabia; afalsharari@ju.edu.sa; 2 Medical Surgical Department, College of Nursing, Jouf University, Sakaka 72388, Saudi Arabia; 3 Faculty of Nursing, King Khalid University, Khamis Mushait 62529, Saudi Arabia; mabuadas@kku.edu.sa

**Keywords:** colorectal cancer, prediction model, screening practices, cancer prevention, screening program

## Abstract

Colorectal cancer (CRC) screening intention is one of the most important elements influencing the longstanding effectiveness of community-based CRC screening programs. The primary purpose of this study is to generate and validate a predictive screening model that investigates the influence of Saudis’ demographics, CRC knowledge, and beliefs on intention to undergo CRC screening via fecal occult blood test (FOBT). Convenience sampling was used to recruit 600 average and high-risk participants from multiple primary health care centers in three major Saudi provinces. A valid and reliable self-administered online survey was used to collect data from March 2021 to October 2021. The final modified screening prediction model explained 57.35% of the variance in screening intention. Intention to screen was significantly influenced by seven factors in which the perceived barriers factor (*β* = −0.55, *p* < 0.001) was the strongest predictor. Those who had lower perceived barriers and greater levels of knowledge (*β* = 0.36, *p* < 0.001), health motivation (*β* = 0.35, *p* < 0.001), perceived benefits of screening (*β* = 0.35, *p* < 0.001), severity (*β* = 0.29, *p* < 0.001), and susceptibility (*β* = 0.28, *p* < 0.001) were more likely to become involved in screening practices. Health care practitioners and various media forms could benefit from the prediction model playing a significant role in raising awareness, reducing perceived barriers, and enhancing Saudi screening rates.

## 1. Introduction

CRC develops as adenomatous benign polyps that grow slowly in the interior layer of the colon or rectum over a period of 10–20 years. The adenomatous polyps grow in size over time and finally transform into cancer cells [1]. The length of this period provides an opportunity to detect and remove precancerous polyps at an early stage. Screening practices of CRC (FOBT and endoscopy) facilitate early detection and thus inhibit CRC Development and improve success rates for CRC treatment [1,2,3]. The fact that CRC generally has no symptoms in its early stages heightens the need for adherence to CRC screening practices [1,2,3]. There are numerous warning signs and symptoms of CRC, such as blood in the stool, black stool, cramping in the lower abdomen, prolonged constipation or diarrhea, decreased appetite, and unintentional weight loss [1,2]. CRC is the world’s second and third most frequent cancer in females and males, respectively [4]. The cancer research agency (GLOBOCAN) predicted more than 1,8 million new cases of CRC and 881 thousand deaths in 2018 [4]. Generally, the incidence rates for CRC are higher in industrialized countries relative to developing countries [4,5,6].

Early and prompt diagnosis of CRC is critical, and the cure rate is very high (90%) [1,2]. On the other hand, a late-stage CRC diagnosis with metastasis to other organs has a 5-year survival probability of about 10% [1,2]. There are two different CRC screening methods: FOBT and endoscopy (colonoscopy and sigmoidoscopy) [7]. Increasing screening via annual high-sensitivity FOBT, colonoscopy, or flexible sigmoidoscopy for the average-risk population aged 40 years or older are the best ways to decrease the CRC mortality rate [1,2]

The Kingdom of Saudi Arabia is a Middle Eastern Arab country with an estimated population of 34,218,169 (recorded in 2018) and has one of the highest fertility rates in the region; it also has a young population (approximately 50% of the population is younger than 25 years of age) [8,9]. Data regarding cancer incidence rates are collected from all 13 administrative regions of Saudi Arabia via the Saudi Cancer Registry (SCR). The SCR continuously collects data from all Ministry of Health hospitals, university hospitals, private hospitals, and clinics. The SCR, which was established in 1992, uses a combination of active (regular visits to hospitals) and passive data collection methods (standard format completion and patient files) to monitor the incidence of cancer in Saudi Arabia over time [10,11].

According to the SCR, there were 15,807 registered cancer cases in Saudi Arabia between 1 January and 31 December 2014, with 7462 men (47.2%) and 8345 (52.8%) women diagnosed. In Saudi Arabia, CRC ranked second among all new cancers diagnosed, and it was the most prevalent cancer in men within the same time period. After breast cancer, it is considered the most prevalent cancer in women. A significant increase in CRC incidence and prevalence was observed in males and females throughout the current period [10,11]. 

In Saudi Arabia, most media emphasis has been concentrated on breast cancer initiatives, with little attention dedicated to CRC [12,13]. Furthermore, in a recent study conducted by Al-Hajeili, Abdulwassi, Alshadadi, Alqurashi, Idriss, and Halawani [12], screening rates for CRC were low in a Saudi adult sample consisting of 422 participants from Jeddah. The results showed that 64% of the Saudi participants knew nothing about CRC and screening options [12]. In another study conducted by Zubaidi, AlSubaie, AlHumaid, Shaik, AlKhayal, and AlObeed [13] in the Riyadh region on an adult sample consisting of 1070 participants, there were some misconceptions regarding CRC knowledge and screening practices among Saudis [13]. 

### Theoretical Framework

The health belief model (HBM) provides the most appropriate theoretical framework within which to investigate the influence of Saudis’ demographics, CRC knowledge, and beliefs on CRC screening practices and intent to undergo CRC screening via FOBT. Godfrey Hochbaum and Stephen Kegels developed the HBM, and Hochbaum originally used it in the early 1950s [14]. The primary constructs of HBM are perceived susceptibility, perceived severity, perceived benefits, perceived barriers, health motivation, cues to action, and self-efficacy [14]. According to the HBM, individuals are more likely to take action to prevent adverse health problems if they perceive themselves to be susceptible to those problems [14,15]. The HBM serves as a framework for motivating people to take positive actions regarding their health, using the desire to avoid negative health consequences as the primary motivation [14,15]. Therefore, the HBM can function as a foundation for the screening prediction model of this study and may accurately explain behavioral modification [14,15].

The HBM has frequently been used to explain and predict cancer screening behavior, but it is rarely used in CRC screening research. The HBM has recognized barriers and predictors, and their relationships to numerous health activities were explained in several studies [14,16]. The HBM was used to examine predictors of CRC screening among the average and high-risk Saudi population in this study. Table 1 illustrates the HBM components and their relationships with the current study variables.

Addressing the Saudi knowledge gap and inappropriate cancer prevention practices could be essential in enabling early detection as a prevention strategy [12]. Further, healthcare providers could play an essential role in developing strategies to increase Saudi knowledge regarding screening tests and changing the health perceptions that may contribute to low screening rates among Saudis [12,17,18]. In Saudi Arabia, there are no screening programs for the average risk and high-risk population who are aged 40 years or more [12,17,18]. It is anticipated that the results of this study can provide a starting point to understand contextual and personal factors that may influence Saudis’ CRC screening practices and intentions to undergo CRC screening. CRC Screening initiatives in Saudi Arabia depend only on the decisions of health care providers and population awareness. Therefore, healthcare providers who deal with average and high-risk Saudi populations in healthcare institutions and community clinics could include assessing these contextual and personal factors (predictors) to increase the uptake of screening practices. In addition, the findings of this study can be utilized to guide the creation of nursing interventions aimed at raising awareness and modifying the health perceptions of the average risk Saudi population regarding CRC and screening options. Improved knowledge and health perceptions about CRC and screening options may also increase Saudis’ participation in early screening for CRC and, consequently, may reduce their incidence and mortality rate. 

The primary purpose of this study is to investigate the influence of Saudis’ demographics, CRC knowledge, and beliefs on CRC screening practices, and intent to undergo CRC screening via FOBT. More specifically, this study focuses on examining CRC screening predictors derived from the HBM. This study answers the following questions: (a) To what extent do the modifying factors (Saudis’ socio-demographics and CRC knowledge) account for a significant portion of the variance in CRC screening practices and intention to undergo screening among the at-risk Saudi population. (b) To what extent do the HBM constructs account for a significant portion of the variance in CRC screening practices and intention to undergo screening among the at-risk Saudi population.

## 2. Materials and Methods

### 2.1. Study Design and Patients 

A cross-sectional descriptive correlational design was used to examine predictors of CRC screening among the Saudi at-risk population. Polit and Beck (2017) state that descriptive correlational research aims to describe the relationship among variables. People aged 40 years or more with no familial CRC history or gastrointestinal signs or symptoms are called the average risk population [1,19]. In contrast, people with a familial CRC history are called high-risk populations [1,19]. The target population was all average-and high-risk Saudi adults. The following eligibility criteria were applied: aged 40–75 years, able to read and write Arabic; able to consent to study participation. The exclusion criteria were a previous CRC diagnosis and inability to consent. The age range was based on screening recommendations from various organizations [1,19]. In addition, a maximum age of 75 years was chosen for sample selection based on the recommendations of the US Preventive Services Task Force against routine screening for adults aged 76–85 years and no screening for adults older than 85 years [19].

### 2.2. Sampling and Setting 

The study sample was recruited from different regions in Saudi Arabia to have a representative sample. Participants were recruited from multiple public health centers in major Saudi cities (Northern, Southern, and central regions). These centers were selected due to the multidisciplinary services they provide to a large proportion of the Saudi population, making it easier to recruit a large sample of people. The study used nonprobability convenience sampling to recruit at-risk Saudi adults who meet the inclusion criteria for the study. According to Woo [20], convenience sampling entails the inclusion of the most conveniently accessible individuals as study participants. The sample size was determined in line with the recommendations of Black and Babin [21], who advised that at least 200 participants were required regardless of the number of observed variables. Moreover, there should be at least 20 cases for each parameter in the analysis. Therefore, 600 participants were approached to participate in the study to increase the opportunity for additional analysis. 

### 2.3. Instruments

Green and Kelly modified the Champion revised HBM scale to explore CRC knowledge, perceptions, and screening behaviors in African American participants. A 57-item scale, the CRCKPSS, was developed to provide a valid and reliable instrument to measure CRC knowledge, perception, and screening behaviors [22]. A modified trans-culturally Arabic version of CRCKPSS was evaluated and validated by factorial analysis of principal components by Abuadas et al. [23] to be used in Arabic culture [23]. The researchers obtained permission to use the modified Arabic version from the original authors. The internal consistency of the modified Arabic version ranged between 0.94 and 0.98. 

The modified Arabic version comprises four core sections. The first section contains 13 sociodemographic questions. The second section contains 13 true or false questions that measure the participant’s knowledge regarding CRC incidence, mortality, signs and symptoms, facts, misconceptions, and screening tests. The number of correctly answered items will determine the total score for each participant. The total score is on an interval level and ranges from 1–13. The third section (perceptions about CRC) consists of four subsections, which measure individual perceived susceptibility to CRC (5 items), perceived severity of CRC (12 items), perceived benefits of screening (5 items), and perceived barriers to screening (13 items). All items in perceptions were measured on a 5-point Likert scale. The fourth section (CRC screening) contains six questions that measure the individual’s CRC screening behavior. Intention to undergo CRC screening via FOBT was measured using one item (a “yes” or “no” scale) [24,25]. 

### 2.4. Data Collection 

The researcher identified potential participants at the primary health care centers according to the eligibility criteria described above. Once participants were identified, the researcher explained the study’s purpose, risks, and benefits to eligible participants. The participants who agreed to participate voluntarily in the study were invited to fill out an online survey. The survey was constructed primarily as an online survey using Qualtrics technology, and it was sent to participants via e-mail and social media. A web link and QR code invitation were sent, and the participants answered the questionnaire online using either a mobile phone or a PC. A few printed survey responses were collected from the participants who could not fill out the online survey. All participants were assigned a code number to ensure anonymity, and all submitted data were handled electronically by the researcher through a password-protected account.

### 2.5. Data Analysis 

Statistical Package for Social Sciences (SPSS) version 25 [26] and Analysis of Moment Structure software (Version 21.0) [27] have been used for all analyses. A descriptive statistical analysis was used to describe frequencies, percentages, means, and standard deviations of the demographic characteristics, measured factors, and variables of participants. We employed a structural equation modeling technique to identify the direct, indirect, and total effects of the affecting factors and construct a hypothetical model. Maximum likelihood estimation has been used for parameter estimation to test the validity of the path, and the following fit indices have been used to determine the fit of the hypothetical model to the data. Pearson’s correlation coefficients estimated the relationships between the variables/factors. Schermelleh-Engel et al. [28] proposed thresholds for an acceptable fit that were utilized as criteria to assess the model fit as follows: (a) factor loadings should have a critical ratio (CR) >1.96, (b) the index of relative chi-square (χ2/df) should be ≤5, (c) the comparative fit index (CFI) and normed fit index (NFI) should be ≥0.85, (d) the goodness of fit index (GFI) and adjusted goodness of fit index (AGFI) should be ≥0.85, and (e) the standardized root mean square residual (RMR) and root mean square error of approximation (RMSEA) should be ≤0.08. Data were checked for independence, normality, and homoscedasticity before the model testing. The case means imputation technique was used to replace random missing data.

### 2.6. Ethical Considerations

The project received ethical approval from Jouf University’s institutional review board (IRB approval no. 09-06-42, Date: 11 March 2021). Several strategies were used to protect the participants’ confidentiality. All participants were made aware that the data gathered would be anonymized, and an informed consent form was attached to the online and printed survey. In addition, all participants were aware of the intended screening test (FOBT) before the beginning of the data collection process.

## 3. Results

### 3.1. Descriptive Statistics

The current study had an 80% response rate, with 600 participants completing online surveys. The average age was 53.25 years, with a standard deviation of 4.36 years. A total of 30.5% were female participants, while 69.5% were male. The majority of the sample underwent secondary education (35.5%). Additionally, 39.2% of those who took part had a family history of CRC. Of those, 30.6% stated that it had affected their grandfather, 24.5% their father, 16.3% their uncle, 10.2% their cousin (father’s side), 8.2% their cousin (mother’s side), and 10.2% other relatives. Furthermore, 67.2% had an income of more than 10,000 Saudi Riyal (SAR) (USD 1 equal to SAR 3.75), and 32.8% had an income of less than SAR 10,000. In total, 86.7% of participants had health insurance coverage. Of those insured, 65% were covered by the governmental sector, 25% by the military sector, and 10% by the private sector (multiple answers were allowed). (See Table 2.) 

Intention to screen for CRC by using FOBT revealed significant moderate correlations with benefits, barriers, severity, susceptibility, motivation, family history and knowledge variables; the strongest correlation was with barriers (r = −0.57, *p* < 0.001), followed by motivations (r = 0.38, *p* < 0.001), benefit (r = −0.36, *p* < 0.01), knowledge (r = 0.36, *p* < 0.001), severity (r = 0.32, *p* < 0.001), and susceptibility (r = 0.31, *p* < 0.001). Weak significant correlations were found between family history and intention to screen (r = 0.12, *p* < 0.01). (See Table 3.)

### 3.2. Testing the Preliminarily Hypothesized Model

**Figure 1:**
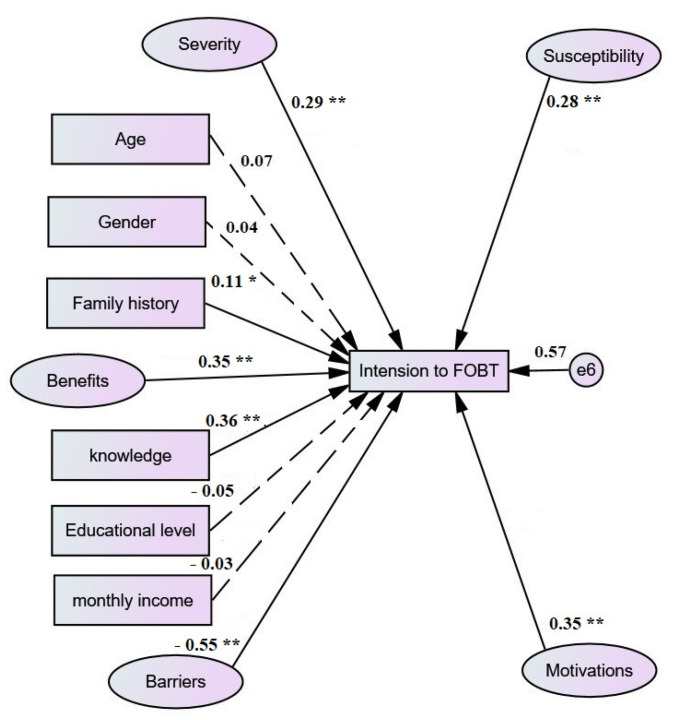
The preliminarily model predicts screening practice intention; dotted lines mean insignificant paths. * *p* < 0.05, ** *p* < 0.001. All regression estimates are standardized *β* coefficients.

The following preliminary hypothesized model (see Figure 1) fit criteria revealed a poor fit: (χ2/df = 2.725, *p* < 0.001, GFI = 0.662, RMSEA = 0.051, AGFI = 0.641, CFI = 0.753). Numerous paths were found non-significant after evaluating modification indices and parameter estimates. Subsequently, they were removed to make the measurement model more theoretically consistent. Age, gender, educational level, and income were also taken out of the equation.

### 3.3. The Modified Stable Prediction Model Testing

**Figure 2:**
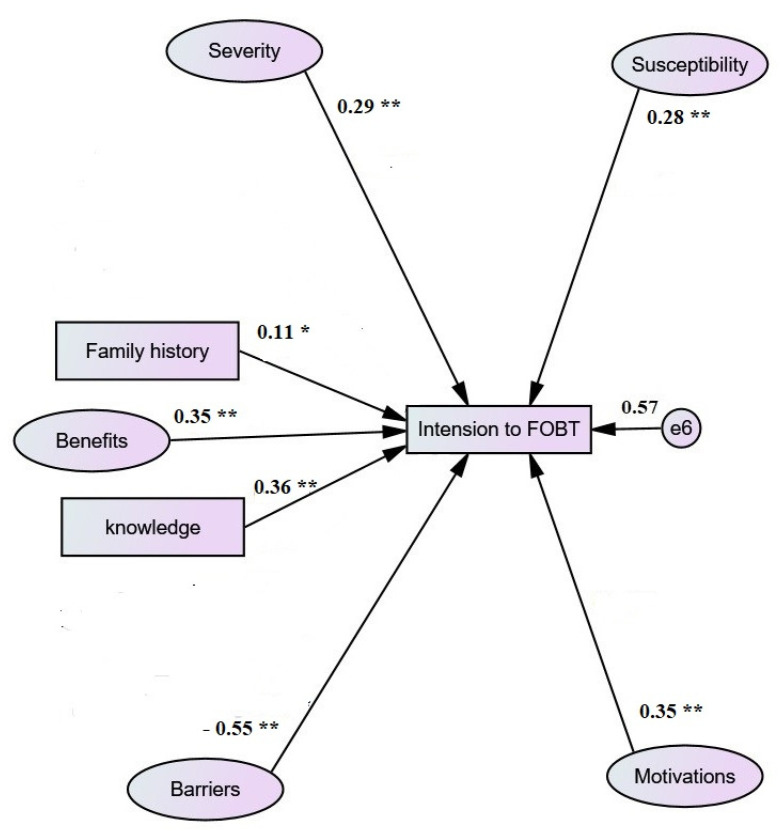
The modified stable model predicting screening practice intention. * *p* < 0.05, ** *p* < 0.001. All regression estimates are standardized *β* coefficients.

The modified stable model shown in Figure 2 has better fit indices than the preliminary model: (χ2/df = 2.26, CFI = 0.90, RMSEA = 0.049, GFI = 0.85, AGFI = 0.83, CFI = 0.91).

### 3.4. Factors Affecting Screening Practice Intention

The influencing factors on intention to screen for CRC using FOBT were specified (Table 3 and Figure 2). Explicitly, the intention to screen was significantly affected by the barriers factor (*β* = −0.55, *p* < 0.001), knowledge (*β* = 0.36, *p* < 0.001), motivations (*β* = 0.35, *p* < 0.001), benefits (*β* = 0.35, *p* < 0.001), severity (*β* = 0.29, *p* < 0.001), susceptibility (*β* = 0.28, *p* < 0.001), and family history (*β* = 0.12, *p* < 0.01). The barriers factor was most strongly related to screening intention. In total, the seven factors explained 57.35% of the variance in screening intention. According to the findings of the study, barriers had a direct positive effect and indirect effect via other factors on screening intention. Greater barriers, in turn, predicted lower screening practice scores. (See Table 4.) 

## 4. Discussion

The participants in this study had comparable sociodemographic and personal characteristics to those prior national and international studies looking at awareness, attitudes, and beliefs about CRC and screening practices [10,13,17,18,29]. However, international studies have revealed a higher educational qualification level. The need for developing a conceptual model to evaluate multiple essential factors related to CRC screening practice implementation arose because most previous literature focused on one or two aspects without considering the complex nature of the implementation [30].

The final prediction model explained 57.35% of the variance in screening intention. In the current study, CRC screening behavior regarding FOBT showed a moderately significant correlation with barriers, benefits, severity, susceptibility, motivation, and knowledge variables. In the modified stable prediction model, perceived barriers were the main predictor of CRC screening behavior. The current study identified embarrassment and fear as the main personal barriers that could decrease CRC screening rates. These results were consistent with national studies in which fear and lack of awareness were the most prevailing barriers among the Saudi population [12,17,31].

In contrast, several studies were conducted to improve understanding of the barriers that maintain low CRC screening rates [32,33,34]. The cost of screening and a lack of access to healthcare (due to having no health insurance) were strongly associated with low screening rates [35]. Similarly, another study conducted by Reyes and Miranda [36] emphasized the importance of healthcare access and health insurance coverage and the role of healthcare providers in improving adherence to CRC screening schedules. Financial constraints are one of the most often mentioned barriers to CRC screening programs. This does not appear to be the case in our study, where government institutions provide health care, and a high percentage of the population is covered by health insurance.

The level of knowledge, health motivation, and perceived benefits, severity, and susceptibility factors showed a moderately significant correlation as predictors for the implementation of CRC screening. Similarly, several researchers [12,17,31,37,38] found a positive and significant correlation between previously mentioned predictors and screening intention. A cross-sectional study with a random sample of 1352 adults (aged 50 years or older) was conducted by Qumseya, Tayem, Dasa, Nahhal, Abu–Limon, Hmidat, Al–Shareif, Hamadneh, Riegert–Johnson, and Wallace [37] to understand the barriers contributing to low CRC screening in Palestine. The results showed the low CRC screening rates among the Palestinian population could be attributed to a lack of CRC awareness among the public and health care providers’ failures to recommend CRC screening. 

Greater perceived susceptibility and severity to CRC has frequently been associated with higher screening uptake [12,17,31,32]. Christou and Thompson [32] conducted a study with a convenience sample of 93 Indigenous Western Australian participants; results indicated that those who perceived higher susceptibility to CRC and greater perceived severity were significantly more likely to consider participation in screening, particularly FOBT. Therefore, there is an increased need to educate and provide accurate information about factors related to perceived susceptibility and severity of CRC, as well as benefits of and barriers to CRC screening practices for the at-risk Saudi population.

An interesting finding in the proposed predictive model was the non-significant predictive effect of gender, age, educational level, and income factors. These results were congruent with some Saudi studies [12,17,39]. However, the level of education and family history in Al-Hajeili, Abdulwassi, Alshadadi, Alqurashi, Idriss, and Halawani [12] were significantly predictive of the status of CRC knowledge. 

Due to the absence of clear CRC screening guidelines and programs in Saudi Arabia [12], health care practitioners and various media types should play an extensive role in improving levels of awareness, decreasing perceived barriers, and enhancing screening rates in the Saudi Arabia population. The current study clarified the influence of Saudis’ demographics, CRC knowledge, and beliefs on CRC screening practices; thus, health care providers and community leaders could use these results to enhance health strategies and target the educational needs of Saudi populations. In addition, this study may provide Saudi nurses with a theoretical-based screening prediction model to be utilized in different health care settings to improve the quality of nursing care and reduce costs. Furthermore, this study may motivate primary health care providers to conduct more research regarding CRC and CRC screening practices.

Given the difficulty in obtaining a well-defined sampling frame, we were unable to collect a random sample from the population, which may restrict the generalizability of the results. Furthermore, all participants were from primary health care centers, which may have features that are not representative of the overall community. However, we attempted to sample different areas to have a more representative sample. The study findings deserve to be replicated with a larger and more heterogeneous sample recruited randomly from different settings. Furthermore, recruiting participants with a CRC family history could affect the proportion of variance in some predictors. Nevertheless, a small proportion of those with a family history were first-degree relatives of CRC patients. 

## Figures and Tables

**Table 1:** Relationship of Health Belief Model components with current study variables.

HBM Construct	Study Variable
Perceived susceptibility	Saudis’ perceived susceptibility to CRC
Perceived severity	Saudis’ perceived severity of CRC
Perceived benefit	Saudis’ perceived benefit of CRC screening
Perceived barriers	Saudis’ perceived barriers to CRC screening
Modifying factors	Demographic factors Age, gender, educational level, income, and health insurance
	Sociopsychological factors Socioeconomic status and family history
	Structural factors Participants’ CRC knowledge
Likelihood of behavioral change	Intent to undergo screening via FOBT Participants’ self-reported intention to undergo FOBT as screening tests for CRC

**Table 2:** Sample Socio-demographics (N = 600).

Variables	N	%
Gender		
Male Female	417 183	69.5% 30.5%
Educational level		
Less than secondary Secondary Diploma Bachelor’s Master	196 213 95 80 16	32.7% 35.5% 15.8% 13.3% 2.6%
Family history		
Yes No	235 365	39.2% 60.8%
Income		
Less than 10,000 SR More than 10,000 SR	197 403	32.8% 67.2%
Insurance		
Yes No	520 80	86.7% 13.3%
Characteristics	Mean ± SD.	
Age (yrs)	53.25 ± 4.36	

SD, standard deviation, % percentage.

**Table 3:** Correlation matrix of main study variables (N = 600).

Variable	1	2	3	4	5	6	7	8	9	10	11	12
Age	1	.										
Gender	0.13 *	1										
Education	0.11 *	−0.02	1									
Family history	0.13 *	−0.02	0.22 **	1								
Income	−0.11 **	−0.09 *	−0.14 **	−0.12 **	1							
Knowledge	0.10	−0.12 **	0.28 **	0.07	−0.13 **	1						
Severity	−0.09 *	−0.12 **	0.13 **	−0.07	−0.09 *	0.08 *	1					
Susceptibility	0.04	0.01	0.22 **	0.13 **	−0.07	0.23 **	0.13 **	1				
Motivation	0.05	−0.06	0.18 **	0.15 **	−0.01	0.23 **	0.13 **	0.16 **	1			
Benefits	−0.04	−0.05	0.26 **	−0.02	−0.07	0.24 **	0.15 **	0.48 **	0.11 **	1		
Barriers	0.02	−0.04	0.01	0.03	0.02	−0.16 **	0.03	−0.35 **	−0.17 **	−0.39 **	1	
Intention to FOBT	0.09	0.06	−0.07	0.12 *	−0.04	0.37 **	0.32 **	0.31 **	0.38 **	0.36 **	−0.57 **	1

* *p* < 0.05; ** *p* < 0.001.

**Table 4:** Direct, indirect, and total effects of variables in the modified stable prediction model (N = 600).

Outcome Variables	Independent Variables	β	Standardized Effects			Squared Multiple Correlations
			Direct Effect	Indirect Effect	Total Effect	
Intention to screen via FOBT	Barriers	−0.55	−0.43 **	0.57	−0.55 **	0.57
	Knowledge	0.36	0.28 **	0.08	0.36 **	
	Motivation	0.35	0.29 **	0.06	0.35 **	
	Benefits	0.35	0.26 **	0.09	0.35 **	
	Severity	0.29	0.25 **	0.04	0.29 **	
	Susceptibility	0.28	0.24 **	0.04	0.28 **	
	Family History	0.11	0.11 *	0	0.11 *	

*p < 0.05; **p < 0.001.

## Data Availability

Not Applicable.

## References

[1] American Cancer Society (2018). Cancer Facts & Figures; The Society: Atlanta, Georgia. https://www.cancer.org/content/dam/cancer-org/research/cancer-facts-and-statistics/annual-cancer-facts-and-figures/2018/cancer-facts-and-figures-2018.pdf.

[2] Siegel R.L., Miller K.D., Jemal A. (2020). Cancer statistics, 2020. CA A Cancer J. Clin..

[3] Mozdiak E., Weldeselassie Y., McFarlane M., Tabuso M., Widlak M.M., Dunlop A., Tsertsvadze A., Arasaradnam R.P. (2019). Systematic review with meta-analysis of over 90 000 patients. Does fast-track review diagnose colorectal cancer earlier?. Aliment. Pharmacol. Ther..

[4] Bray F., Ferlay J., Soerjomataram I., Siegel R.L., Torre L.A., Jemal A. (2018). Global cancer statistics 2018: GLOBOCAN estimates of incidence and mortality worldwide for 36 cancers in 185 countries. CA A Cancer J. Clin..

[5] Stewart B., Wild C.P. (2014). World Cancer Report 2014.

[6] Ferlay J., Soerjomataram I., Ervik M., Dikshit R., Eser S., Mathers C., Rebelo M., Parkin D., Forman D., Bray F. (2013). GLOBOCAN 2012 v1. 0, Cancer Incidence and Mortality Worldwide: IARC CancerBase No. 11. globocan.iarc.fr2014.

[7] Li J.N., Yuan S.Y. (2019). Fecal occult blood test in colorectal cancer screening. J. Dig. Dis..

[8] Statistics H. (2018). General Authority for statistics: Kingdom of Saudi Arabia. https://database.stats.gov.sa/beta/dashboard/landing.

[9] General Authority for Statistics (2018). Population & Demography. Statistical Yearbook of 2018. https://www.stats.gov.sa/en/1007-0.

[10] Alsanea N., Abduljabbar A.S., Alhomoud S., Ashari L.H., Hibbert D., Bazarbashi S. (2015). Colorectal cancer in Saudi Arabia: Incidence, survival, demographics and implications for national policies. Ann. Saudi Med..

[11] Al-Shahrani Z., Al-Rawaji A., Al-Madouj A., Hayder M. (2017). Saudi Cancer Registry: Cancer Incidence Report Saudi Arabia 2014. Saudi Health Counc. Riyadh Saudi Arab..

[12] Al-Hajeili M., Abdulwassi H.K., Alshadadi F., Alqurashi L., Idriss M., Halawani L. (2019). Assessing knowledge on preventive colorectal cancer screening in Saudi Arabia: A cross-sectional study. J. Fam. Med. Prim. Care.

[13] Zubaidi A.M., AlSubaie N.M., AlHumaid A.A., Shaik S.A., AlKhayal K.A., AlObeed O.A. (2015). Public awareness of colorectal cancer in Saudi Arabia: A survey of 1070 participants in Riyadh. Saudi J. Gastroenterol. Off. J. Saudi Gastroenterol. Assoc..

[14] Champion V.L., Skinner C.S. (2008). The Health Belief Model. Health Behaviour and Health Education; Theory, Research, and Practice.

[15] Glanz K., Rimer B.K., Viswanath K. (2008). Health Behavior and Health Education: Theory, Research, and Practice.

[16] Rimer B.K. (2008). Models of individual health behavior. Health Behav..

[17] Almadi M.A., Mosli M.H., Bohlega M.S., Al Essa M.A., AlDohan M.S., Alabdallatif T.A., AlSagri T.Y., Algahtani F.A., Mandil A. (2015). Effect of public knowledge, attitudes, and behavior on willingness to undergo colorectal cancer screening using the health belief model. Saudi J. Gastroenterol. Off. J. Saudi Gastroenterol. Assoc..

[18] Almutairi K.M., Alonazi W.B., Alodhayani A., Vinluan J.M., Ahmad M., Alhurishi S.A., Alsadhan N., Alsalem M.M., Alotaibi N.E., Alaqeel A.M. (2018). A cross-sectional assessment of literacy and awareness, attitudes, and beliefs about colorectal cancer and its screening in Riyadh Region. J. Cancer Educ..

[19] Smith R.A., Cokkinides V., Brooks D., Saslow D., Brawley O.W. (2010). Cancer screening in the United States, 2010: A review of current American Cancer Society guidelines and issues in cancer screening. CA A Cancer J. Clin..

[20] Woo K. (2017). Polit & Beck Canadian Essentials of Nursing Research.

[21] Black W., Babin B.J. (2019). Multivariate data analysis: Its approach, evolution, and impact. The Great Facilitator.

[22] Green P.M., Kelly B.A. (2004). Colorectal cancer knowledge, perceptions, and behaviors in African Americans. Cancer Nurs..

[23] Abuadas F.H., Abuadas M.H., Alsharari A.F., Albikawi Z.M. (2021). Translation, Trans-Cultural Adaptation to Arabic, and Psychometric Testing of a Questionnaire Measuring Colorectal Cancer Knowledge, Perceptions, and Screening Practices among Average-Risk Population. Asian Pac. J. Cancer Prev.

[24] Griffin M.J. (2011). Health Belief Model, Social Support, and Intent to Screen for Colorectal Cancer in Older African American Men. Ph.D. Thesis.

[25] Sieverding M., Matterne U., Ciccarello L. (2010). What role do social norms play in the context of men’s cancer screening intention and behavior? Application of an extended theory of planned behavior. Health Psychol..

[26] Corportation I. (2017). IBM SPSS Statistics for Windows (Version 25.0 Armonk).

[27] Arbuckle James L. (2012). IBM SPSS Amos 21 User’s Guide.

[28] Schermelleh-Engel K., Moosbrugger H., Müller H. (2003). Evaluating the fit of structural equation models: Tests of significance and descriptive goodness-of-fit measures. Methods Psychol. Res. Online.

[29] Abuadas F.H., Petro-Nustas W.J., Abuadas M.H. (2018). The effect of a health education intervention on Jordanian participants’ colorectal cancer knowledge, health perceptions, and screening practices. Cancer Nurs..

[30] Cheng L., Feng S., Hu Y. (2017). Evidence-based nursing implementation in mainland China: A scoping review. Nurs. Outlook.

[31] Galal Y.S., Amin T.T., Alarfaj A.K., Almulhim A.A., Aljughaiman A.A., Almulla A.K., Abdelhai R.A. (2016). Colon cancer among older Saudis: Awareness of risk factors and early signs, and perceived barriers to screening. Asian Pac. J. Cancer Prev..

[32] Christou A., Thompson S.C. (2012). Colorectal cancer screening knowledge, attitudes and behavioural intention among Indigenous Western Australians. BMC Public Health.

[33] Paddison J.S., Yip M.J. (2010). Exploratory study examining barriers to participation in colorectal cancer screening. Aust. J. Rural. Health.

[34] Huang J., Choi P., Pang T.W., Chen X., Wang J., Ding H., Jin Y., Zheng Z.J., Wong M.C. (2021). Factors associated with participation in colorectal cancer screening: A population-based study of 7200 individuals. Eur. J. Cancer Care.

[35] Holden D.J., Jonas D.E., Porterfield D.S., Reuland D., Harris R. (2010). Systematic review: Enhancing the use and quality of colorectal cancer screening. Ann. Intern. Med..

[36] Reyes A.M., Miranda P.Y. (2015). Trends in cancer screening by citizenship and health insurance, 2000–2010. J. Immigr. Minority Health.

[37] Qumseya B.J., Tayem Y.I., Dasa O.Y., Nahhal K.W., Abu–Limon I.M., Hmidat A.M., Al–Shareif A.F., Hamadneh M.K., Riegert–Johnson D.L., Wallace M.B. (2014). Barriers to colorectal cancer screening in palestine: A national study in a medically underserved population. Clin. Gastroenterol. Hepatol..

[38] Rawl S.M., Skinner C.S., Perkins S.M., Springston J., Wang H.-L., Russell K.M., Tong Y., Gebregziabher N., Krier C., Smith-Howell E. (2012). Computer-delivered tailored intervention improves colon cancer screening knowledge and health beliefs of African-Americans. Health Educ. Res..

[39] García A.Z.G., Quintero E., Pérez D.N., Hernández M., JiménezSosa A. (2011). Colorectal cancer screening in first-degree relatives of colorectal cancer: Participation, knowledge, and barriers against screening. Eur. J. Gastroenterol. Hepatol..

